# Three-Dimensional (3D) Analysis of Orbital Morphometry in Healthy Anatolian Adults: Sex, Side Discrepancies, and Clinical Relevance

**DOI:** 10.7759/cureus.45208

**Published:** 2023-09-14

**Authors:** Hüseyin Erdem, Mustafa Tekeli, Yigit Cevik, Nazire Kilic Safak, Omer Kaya, Neslihan Boyan, Ozkan Oguz

**Affiliations:** 1 Department of Anatomy, Cukurova University, Faculty of Medicine, Adana, TUR; 2 Department of Radiology, Cukurova University, Faculty of Medicine, Adana, TUR

**Keywords:** orbital opening, orbit, morphometric analysis, 3d reconstruction, 3d analysis

## Abstract

Background and objective

The complex structural integrity of the orbit is crucial for visual functions. Three-dimensional (3D) reconstructions from radiological images have revolutionized anatomical understanding, eliminating the limitations of two-dimensional (2D) imaging and offering intricate spatial details for complex structures. The aim of this study was to analyze the detailed morphometry of the orbit in healthy Anatolian adults, considering sex, side discrepancies, and clinical relevance using 3D models reconstructed from multidetector computed tomography (MDCT) images.

Materials and methods

Fifty-six (44.44%) males and 70 (55.56%) females (total: 126, mean age: 48.62) MDCT images were randomly selected and 3D skull models were reconstructed using 3D Slicer software. Measurements were conducted in millimeters (mm) for transverse and vertical diameters, circumference of the orbital opening, orbital wall lengths, as well as intraorbital and extraorbital distances.

Results

The method of measurements showed high reproducibility of results. The mean values for transverse and vertical diameters, circumference of the orbital opening, medial and lateral wall lengths, roof and floor lengths, and intraorbital and extraorbital distances were 40.23±2.12 mm (p<0.001), 34.94±2.16 mm (p=0.365), 44.74 ±3.02 mm (p<0.001), 46.30±2.69 mm (p<0.001), 51.26±2.91 mm (p<0.001), 49.01±3.22 mm (p<0.001), 126.10±5.71 mm (p<0.001), 19.63±2.35 mm (p=0.026), and 94.09±4.84 mm (p<0.001), respectively.

Conclusion

The study's high measurement reproducibility contributes significantly to the existing literature and clinical practice. These findings offer specific insights into Anatolian orbital morphometry, aiding in surgical planning, implant placement, and diagnostic assessments. The precise measurement values serve as a reliable reference for clinicians, facilitating the identification of normal and abnormal orbital anatomy and enhancing patient care. We believe this study provides valuable data for craniofacial and ophthalmological research, benefiting both clinical practice and future research endeavors in these fields.

## Introduction

The orbit is a pyramidal-shaped cavity formed by certain cranial bones and houses the eyeball along with its appendages [1]. The structural integrity of the orbit is vital for optimal visual functions. Any dysmorphia in its structure can lead to displacement of the eyeball and result in exophthalmos, enophthalmos and diplopia [1]. The morphology and the size of the orbit can be affected by various conditions, such as Crouzon syndrome and Graves' disease and these morphological changes can provide valuable diagnostic clues [2,3]. Early detection and effective management of these conditions can be achieved by utilizing advanced diagnostic tools which are essentially guided by comprehensive anatomical knowledge of the orbital morphology [4].

Digital 3D reconstruction techniques have emerged as transformative methods. These applications have been adapted to medical sciences to understand or describe the anatomical structures and identify any condition, from trauma to malformations [5,6]. Traditional 2D imaging techniques are inherently inadequate for accurately assessing complex anatomical structures, such as the orbit [7]. The introduction of 3D reconstructions from Digital Imaging and Communications in Medicine (DICOM) datasets has been revolutionary in terms of providing intricate information regarding the spatial relationships, depth, and precise measurements of complex anatomical structures [8].

Consideration of the morphometric data pertaining to the orbital dimensions is crucial in a range of clinical applications, which span from the diagnosis and management of diseases to planning surgical interventions for decompressive or reconstructive procedures [1,9]. However, the existing literature on orbital morphometrics is limited to a few studies that lack comprehensive, region-specific 3D data in the Anatolian population [10-13].

The objective of this study is to provide a detailed exploration of the orbital morphometrics by analyzing healthy Anatolian adults using 3D models reconstructed from DICOM datasets to provide an accurate representation of the relevant area.

## Materials and methods

This retrospective study received approval from the local non-interventional clinical research ethics committee (Protocol no: 5.4.21/38-109) and adhered to the principles outlined in the Declaration of Helsinki. The dataset for the current study consisted of axial multidetector computed tomography (MDCT) images of patients screened between January 2021 and December 2021. A randomized approach was employed to select MDCT images from the archive of the Radiology Department. All data utilized for the study were anonymized to uphold the confidentiality of patient information.

**Figure 1:**
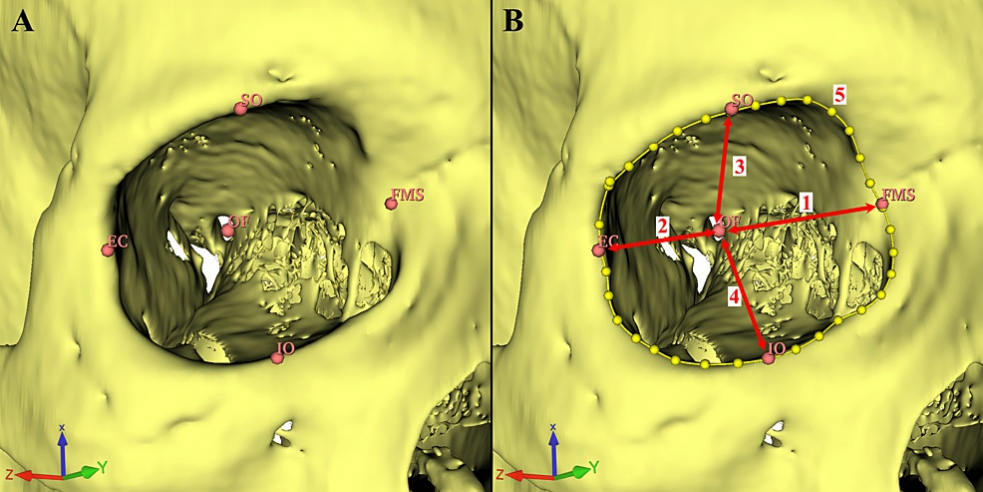
Definition of the anatomical landmarks. A. Frontomaxillary suture (FMS): The point of intersection between FMS and the medial margin of the orbit. Ectoconchion (EC): Intersection between the lateral orbital rim and the line bisecting the orbital opening into two equal halves. Supraorbital point (SO): Intersection between the line bisecting the FMS-EC and the superior orbital rim. Infraorbital point (IO): Intersection between the line bisecting the FMS-EC and the inferior orbital rim. Optic foramen (OF): The opening of the optic canal to the middle cranial fossa. B. 1: Medial orbital wall length (FMS-OF); 2: Lateral orbital wall length (EC-OF); 3: Orbital roof length (SO-OF); 4: Orbital floor length (IO-OF); 5: Circumference of the orbital opening (dotted yellow line surrounding the orbital opening) (OC) [1].

**Figure 2:**
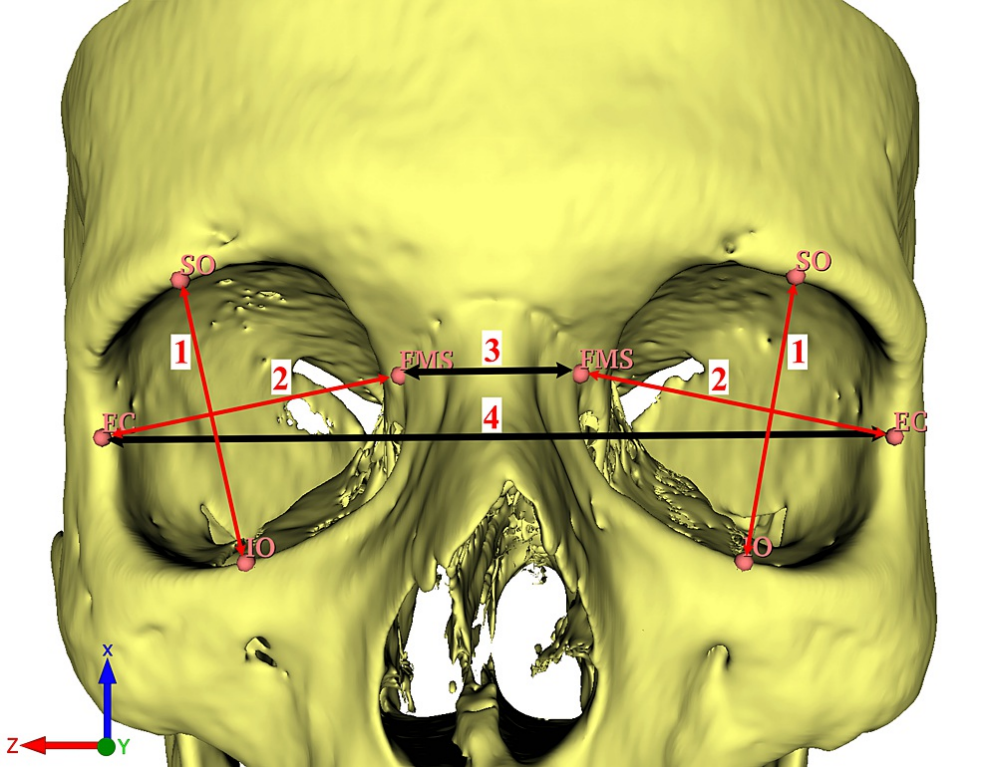
Vertical, transverse, intraorbital and extraorbital distances. 1: The vertical diameter was measured as the distance between the SO and IO. 2: The transverse diameter was measured as the distance between the EC and FMS. 3: The intraorbital distance referred to the measurement between bilateral FMS landmarks. 4: The extraorbital distances were determined by measuring the distance between bilateral EC landmarks [1]. FMS: Frontomaxillary suture; EC: Ectoconchion; SO: Supraorbital point; IO: Infraorbital point

The scans were conducted using a 160-slice MDCT scanner (Aquilion™ PRIME; Toshiba, Otawara, Japan) with a standard protocol of 0.6 mm collimation, 0.5 mm slice thickness, 120 kV, and 250 m. A bone window setting (Width: 2500; Level: 500) and a digital workstation (Vitrea CT workstation; Toshiba) were used to evaluate the images. A series of 179 MDCT images were selected. After applying exclusion criteria (orbital trauma, nasal trauma or deformation, congenital orbital anomalies, patients with surgical history in the head and neck region, or any conditions that could potentially influence the orbital structure) 126 MDCT images (male: 56, age: 47.05±18.30; female: 70, age: 49.87±16.19) were left and used in the study. Segmentation and morphometric measurements were performed using the 3D Slicer software, an open-source software platform, according to the previously described protocol [14,15]. Anatomical landmarks and the morphometric measurements are shown in Figures 1, 2. All measurements were repeated three times and averaged. The morphometric length parameters of the orbit and their definitions are listed in Table 1.

**Table 1:** Morphometric length parameters of the orbit and their definitions. FMS: Frontomaxillary suture; EC: Ectoconchion; SO: Supraorbital point; IO: Infraorbital point; OF: Optic foramen

Parameters	Definitions
Transverse diameter	FMS-EC
Vertical diameter	SO-IO
Medial orbital wall length	FMS-OF
Lateral orbital wall length	EC-OF
Orbital roof length	SO-OF
Orbital floor length	IO-OF
Circumference of the orbital opening	OC
Intraorbital distance	Left FMS - Right FMS (LFMS-RFMS)
Extraorbital distance	Left EC - Right EC (LEC-REC)

Statistical analysis

The IBM SPSS Statistics Version 20.0 (IBM Corp., Armonk, NY, USA) was used for the statistical analysis of the data. Continuous variables were represented as both mean values with standard deviation and as median values with minimum-maximum ranges. Distribution categorization of the datasets into parametric and nonparametric groups was conducted utilizing the Kolmogorov-Smirnov test. A comparative analysis of the morphometric variables between the male and female groups was performed using the Mann-Whitney U test. The correlation analysis between the parameters was evaluated by employing Spearman's rank correlation coefficient, whereas the Wilcoxon rank-sum test was used to analyze the bilateral parameters. Inter-observer reliability was analyzed by means of intra-class correlation coefficients (ICC). P-values <0.05 were considered to be statistically significant.

## Results

The ICCs for each of the measurements are shown in Table 2. According to the evaluation method, inter-observer reliabilities of the repeated measures were good and excellent [16].

**Table 2:** Intra-observer reproducibility and reliability for each the measurements taken. ICC: Intraclass Correlation Coefficient, CI: confidence interval

Parameters	ICC (95% CI)	p
Right transverse diameter (mm)	0.927 (0.896-0.948)	<0.001
Left transverse diameter (mm)	0.928 (0.898-0.949)	<0.001
Right vertical diameter (mm)	0.942 (0.917-0.959)	<0.001
Left vertical diameter (mm)	0.948 (0.926-0.964)	<0.001
Right medial wall (mm)	0.867 (0.811-0.907)	<0.001
Left medial wall (mm)	0.887 (0.839-0.920)	<0.001
Right lateral wall (mm)	0.896 (0.853-0.927)	<0.001
Left lateral wall (mm)	0.847 (0.783-0.893)	<0.001
Right roof (mm)	0.866 (0.809-0.906)	<0.001
Left roof (mm)	0.886 (0.837-0.920)	<0.001
Right floor (mm)	0.814 (0.735-0.869)	<0.001
Left floor (mm)	0.838 (0.770-0.886)	<0.001
Right circumference (mm)	0.964 (0.49-0.975)	<0.001
Left circumference (mm)	0.978 (0.968-0.984)	<0.001
Intraorbital distance (mm)	0.933 (0.904-0.953)	<0.001
Extraorbital distance (mm)	0.903 (0.862-0.932)	<0.001

This study utilized DICOM datasets from 126 individuals (56 males, 70 females). The mean age was 48.62±17.15 years (male: 47.05±18.30; female: 49.87±16.19) with a range of 18 to 85 years. No statistically significant differences were observed in the age distribution between males and females (p=0.365). However, all measurements, with the exception of vertical diameter, were significantly different between males and females (Table 3). Significant differences were observed for vertical diameter (p<0.001), lateral wall length (p=0.005) and orbital roof lengths (p<0.001) between the left and right sides (Table 4). There were high positive correlations between transverse diameter and circumference and extraorbital distance; between medial wall and roof; between lateral wall and roof and floor; between roof and floor; and between circumference and extraorbital distance (Table 5).

**Table 3:** Distribution of male and female morphometric measurements. Values are given as mean±standard deviation and median (min-max). ϯ These values indicate statistical significance (p<0.05).

Variables	Male	Female	Total	p value
Age	47.05±18.30 46.00 (18.00-85.00)	49.87±16.19 49.00 (18.00-84.00)	48.62±17.15 48.00 (18.00-85.00)	0.365
Transverse diameter (mm)	41.34±2.32 41.64 (33.71-44.81)	39.34±1.44 39.19 (35.12-42.66)	40.23±2.12 40.18 (33.71-44.81)	<0.001^ϯ^
Vertical diameter (mm)	34.71±2.00 34.67 (30.26-39.38)	35.12±2.28 35.36 (31.04-40.44)	34.94±2.16 35.07 (30.26-40.44)	0.365
Medial wall (mm)	46.41±2.78 46.45 (40.69-52.31)	43.40±2.51 43.62 (38.24-50.38)	44.74±3.02 44.61 (38.24-52.31)	<0.001^ϯ^
Lateral wall (mm)	47.84±2.52 47.94 (42.59-55.31)	45.07±2.13 44.98 (40.82-49.91)	46.30±2.69 46.07 (40.82-55.31)	<0.001
Roof (mm)	52.88±2.48 53.11 (46.70-57.89)	49.96±2.56 49.87 (45.24-54.79)	51.26±2.91 51.42 (45.24-57.89)	<0.001^ϯ^
Floor (mm)	50.50±3.03 50.16 (42.60-57.40)	47.82±2.88 47.49 (42.93-55.64)	49.01±3.22 48.81 (42.60-57.40)	<0.001^ϯ^
Circumference (mm)	127.81±5.73 128.66 (107.34-139.26)	124.73±5.35 125.39 (108.86-137.50)	126.10±5.71 126.62 (107.34-139.26)	<0.001^ϯ^
Intraorbital distance (mm)	20.14±2.56 20.44 (14.58-24.78)	19.22±2.10 19.37 (15.46-24.58)	19.63±2.35 19.68 (14.58-24.78)	0.026^ϯ^
Extraorbital distance (mm)	96.15±5.34 97.51 (75.67-104.96)	92.44±3.68 92.58 (83.39-100.49)	94.09±4.84 93.90 (75.67-104.96)	<0.001^ϯ^

**Table 4:** Distribution of left and right side morphometric measurements. Values are given as mean±standard deviation and median (min-max). ϯ These values indicate statistical significance (p<0.05).

Variables	Left	Right	p value
Transverse diameter (mm)	40.13±2.27 39.83 (33.42-46.42)	40.33±2.18 40.26 (33.99-47.15)	0.142
Vertical diameter (mm)	35.41±2.13 35.50 (30.67-41.36)	34.46±2.13 34.47 (29.60-39.60)	<0.001^ϯ^
Medial wall (mm)	45.02±2.93 44.99 (39.10-50.87)	44.82±3.33 44.79 (37.47-54.97)	0.193
Lateral wall (mm)	46.31±2.65 46.29 (41.92-54.56)	46.55±3.06 46.01 (40.08-56.06)	0.005^ϯ^
Roof (mm)	51.36±2.58 51.33 (45.60-56.11)	51.50±3.28 51.33 (42.33-59.93)	<0.001^ ϯ^
Floor (mm)	49.51±3.26 49.70 (42.29-56.11)	48.71±3.52 48.39 (42.91-58.81)	0.173
Circumference (mm)	125.94±6.70 126.12 (107.41-139.87)	125.86±5.53 126.31 (107.26-138.64)	0.100

**Table 5:** Spearman correlation coefficients (r) between pairs of parameters. *. Correlation is significant at the 0.05 level (2-tailed). **. Correlation is significant at the 0.01 level (2-tailed).

	Age	Transverse diameter	Vertical diameter	Medial wall	Lateral wall	Roof	Floor	Circumference	Intraorbital distance
Transverse diameter	.045								
Vertical diameter	-0.023	0.179^*^							
Medial wall	0.003	0.524^**^	-0.178^*^						
Lateral wall	0.179^*^	0.506^**^	0.017	0.654^**^					
Roof	-0.068	0.398^**^	-0.034	0.775^**^	0.763^**^				
Floor	0.240^**^	0.477^**^	0.036	0.672^**^	0.880^**^	0.711^**^			
Circumference	0.028	0.736^**^	0.607^**^	0.294^**^	0.481^**^	0.398^**^	0.492^**^		
Intraorbital distance	0.178^*^	0.271^**^	0.027	0.270^**^	0.283^**^	0.321^**^	0.344^**^	0.244^**^	
Extraorbital distance	0.182^*^	0.770^**^	0.250^**^	0.430^**^	0.590^**^	0.436^**^	0.565^**^	0.708^**^	0.674^**^

## Discussion

The present study provides a comprehensive morphometric analysis of the orbit in a healthy Anatolian population, revealing significant sex and left-right side disparities. Transverse and vertical diameters, orbital wall lengths, circumferences, and intraorbital and extraorbital distances are the metrics included in the study obtained from 3D digital craniometric models. Another aspect of this study was the comparative analysis of our findings with similar data reported in previous literature. It is remarkable that the approaches used in these comparative investigations are diverse, ranging from simple dry bone examinations to highly advanced CT and 3D modeling methods.

In this study, all parameters, except the vertical diameter, were significantly larger in males compared to females. The disparity of the orbital opening diameters could be clinically important in the surgical planning of orbital decompression procedures in patients with Graves' ophthalmopathy, or repair of orbital fractures [3,17]. Furthermore, the significant differences in orbital circumferences between sexes are particularly important, as variations in orbital volume have been linked to a number of clinical conditions and disorders. These include age-related shrinkage of orbital fat, orbital fractures, and thyroid eye disease [2]. The morphometric differences in intraorbital and extraorbital distances and orbital wall lengths suggest that preoperative surgical planning for such procedures should take sex-specific characteristics into account. It may be necessary to modify surgical methods and tools to account for sex-based morphometric variances [18].

This study revealed bilateral symmetry and significant differences between the left and right orbits which could have potential clinical implications. While the transverse diameter, medial wall, orbital floor, and circumference lengths showed no significant differences between the left and right orbits, we observed notable asymmetry in the vertical diameter, the length of the lateral wall, and the roof of the orbit. Orbital asymmetry findings can be utilized in the management of orbital fractures and ocular prosthetics design [19]. It could also play a crucial role in monitoring and designing of therapies for diseases such as thyroid eye disease and orbital tumors [2].

The high positive correlations observed among the orbital parameters in our study may have significant clinical implications. The information regarding the relationship between the lateral wall and the floor lengths could assist in diagnosing the potential orbital floor fractures, often observed in facial trauma. Similarly, the highly correlated dimensions of the roof and the medial and lateral walls might be practical in identifying congenital defects, traumatic injuries, or neoplastic formations affecting the orbits or eyeball [4,20]. Additionally, the correlations between orbital circumference and transverse and vertical diameters could be utilized to evaluate overall orbital anatomy, which is critical in surgical planning for procedures such as orbital decompression [21].

Comparison of our results with related studies from the literature revealed similarities as well as differences in orbital morphometry (Table 6). Ji et al. conducted a 3D model study on a Chinese population and reported narrower dimensions for the orbits compared to our findings [1]. Similarly, Mani et al. conducted a 3D model study that also reported narrower orbital dimensions in contrast to our findings [22]. These differences could be attributed to ethnic variations between populations.

**Table 6:** Comparison of our results with similar studies from the literature. CBCT: cone beam computed tomography, MDCT: multidetector computed tomography

_Studies and Methodologies_	_Transverse diameter (mm)_				_Vertical diameter (mm)_				_Medial orbital wall length (mm)_						_Lateral orbital wall length (mm)_					_Orbital roof length (mm)_		_Orbital floor length (mm)_		_Circumference of the orbital opening (mm)_	_Intraorbital distance (mm)_		_Extraorbital distance (mm)_	
Current study 3D models	Total				Total				Total						Total					Total		Total		Total	Total		Total	
	40.23				34.94				44.74						46.30					51.26		49.01		126.10	19.63		94.09	
Mani et al. [20] 3D models	Male		Female		Male		Female		Male		Female				Male		Female			Male	Female	Male	Female		Male	Female	Male	Female
	29.80		29.47		23.38		21.32		5.31		3.50				27.41		23.35			15.26	12.24	13.58	11.88		22.48	19.82	78.23	74.22
Ji et al. [1] 3D models	Male		Female		Male		Female		Male		Female				Male		Female			Male	Female	Male	Female		Male	Female	Male	Female
	40.02		38.00		40.02		38.00		46.43		44.41				48.38		46.91			51.84	51.67	47.93	46.18					
	Left		Right		Left		Right		Left		Right				Left		Right			Left	Right	Left	Right		27.18	25.11	98.77	93.69
	38,94		39.10		38,94		39.10		45.36		45.18				47.60		47.77			52.93	50.89	47.00	46.85					
Calguner et al. [8] Dry bones	Male		Female		Male		Female																					
	35.7		34.4		33.0		32.7																					
Sangvichien et al. [20] Dry bones	Male		Female																									
	40.1		38.09																									
Nitek et al. [7] Dry bones	Male		Female		Total				Male						Female													
	42.6		40.3		33.5				Left			Right			Left			Right										
									42.1			42.6			39.9			40										
Ghorai et al. [22] X-ray	Male		Female		Male		Female																					
	Left	Right	Left	Right	Left	Right	Left	Right																				
	32.79	32.36	31.65	30.81	28.54	28.51	33.23	28.69																				
Sinanoglu et al. [10] CBCT	Male		Female		Male		Female																					
	Left	Right	Left	Right	Left	Right	Left	Right																				
	37.3	38.3	30.2	29.7	40.3	40.3	33.5	34.3																				
Weaver et al. [25] CT					Male		Female																					
					32.44		31.75																					
Kaya et al. [24] CT	Total				Total																							
	Left		Right		Left		Right																					
	36.45		33.18		33.3		33.8																					
Ozer et al. [9] CT	Male		Female		Male		Female																					
	Left	Right	Left	Right	Left	Right	Left	Right																				
	33.99	37.17	33.07	33.27	37.7	37.77	36.55	36.97																				
Attia et al. [23] MDCT	Male		Female		Male		Female																					
	Left	Right	Left	Right	Left	Right	Left	Right																				
	37.2	36.7	37.1	37	37.3	36.9	35.9	35.9																				
El-Farouny et al. [26] MDCT	Male		Female		Male		Female																					
	Left	Right	Left	Right	Left	Right	Left	Right																				
	36.79	37.03	37.39	36.11	34.92	35.18	35.08	34.77																				
Pirinc B et al. [11] MDCT	Male		Female		Male		Female		Male				Female		Male			Female										
	Left	Right	Left	Right	Left	Right	Left	Right	Left	Right			Left	Right	Left	Right		Left	Right									
	32.87	32.97	31.77	31.76	36.64	36.96	35.03	35.22	38.68	38.5			37.72	37.58	42.7	42.72		41.31	40.94									

Other studies included in the comparison utilized different methodologies and patient populations. Calguner [10] and Sangvichien et al. [23] conducted dry bone studies, while Ghorai et al. [24] used X-ray images. These studies provided limited measurements and therefore it is not practical to apply direct comparisons with our findings. Similarly, Nitek et al. [9] reported measurements on dry bones, which may not fully reflect in vivo conditions. Studies utilizing dry bones presented relatively lower values than ours, which may be related to the shrinkage of the bones in the drying process.

Studies using cone beam computed tomography (CBCT) [12] and CT scans [11,13,25-28] reported variable transverse and vertical diameters and also intraorbital distances compared to our results. These variations can be affected by differences in imaging protocols, slice thicknesses, and patient characteristics such as age and sex.

Limitations

Limitations of this study include the lack of functional assessment, difficulties in comparing methodologies with previous studies, the absence of exploration of underlying factors, and the cross-sectional nature of the data. Further research is needed to address these limitations and provide a more comprehensive understanding of orbital morphometry.

## Conclusions

In conclusion, this study provides a thorough examination of orbital morphometry in a healthy Anatolian population and identifies important sex and left-right side discrepancies. Our results may have important clinical implications for surgical planning in procedures such as orbital decompression and orbital fracture repair. Additionally, this study highlights the potential impact of orbital asymmetry in the management of fractures, ocular prosthetics design, and the treatment of diseases such as thyroid eye disease and orbital tumors. The observed correlations among orbital parameters offer valuable diagnostic information for identifying fractures, congenital defects, and neoplastic formations. Comparison with similar studies points out the influence of ethnic variations, methodologies, and imaging techniques on orbital measurements. These findings emphasize the need for considering population-specific characteristics and standardized measurement techniques in clinical practice.
